# Dietary supplementation of protocatechuic acid improves antioxidant capacity and intestinal health in late-laying hens

**DOI:** 10.3389/fmicb.2026.1866286

**Published:** 2026-09-22

**Authors:** Jiahao Yan, Kaiqi Weng, Yang Yang, Tiantian Gu, Tao Zhang, Xin Huang

**Affiliations:** 1 Institute of Animal Husbandry and Veterinary Science, Zhejiang Academy of Agricultural Sciences, Hangzhou, China; 2 Institute of Animal Science and Veterinary Medicine, Shanghai Academy of Agricultural Sciences, Shanghai, China; 3 College of Animal Science and Technology, Yangzhou University, Yangzhou, China; 4 Wowo Agriculture Co., Ltd, Wenzhou, China

**Keywords:** antioxidant capacity, feed supplement, intestinal health, late-laying hen, protocatechuic acid

## Abstract

A decline in the antioxidant capacity of the organism is observed in late-laying hens. Protocatechuic acid (PCA), a phenolic compound, possesses antioxidant and anti-inflammatory properties. However, whether dietary supplementation with PCA can alleviate these adverse effects in late-laying hens remains unclear. A total of 576 Hy-Line Brown laying hens aged 500 days were randomly divided into 4 groups, each with 6 replicates of 24 hens. The four groups consisted of a basal diet group (CK) and three experimental groups supplemented with 0.05, 0.1, and 0.2% PCA (LPCA, MPCA, and HPCA, respectively). The trial lasted 49 days, including a 1-week adaptation period followed by a 6-week experimental period. Dietary PCA supplementation significantly increased the activities of superoxide dismutase (SOD), catalase (CAT), and glutathione peroxidase (GSH-Px) and decreased the level of malondialdehyde (MDA). Furthermore, PCA supplementation also significantly decreased the levels of inflammatory factors such as tumor necrosis factor-alpha (TNF-α), interleukin-1 beta (IL-1β), and interleukin-6 (IL-6). Ileum transcriptomic analyses revealed that the differentially expressed genes were significantly enriched in pathways such as transmembrane transport activity, signal receptor binding, the PPAR signaling pathway, and intestinal immunity. We further sequenced cecal microbiota, showing that the structure of the microbiota has undergone significant changes; beneficial bacterial communities such as g_Bacteroides and g_Odoribacter were significantly enriched in the PCA treatment group. Metabolomic analysis revealed that the differential metabolites were predominantly enriched in pathways closely associated with intestinal digestive and absorptive functions, including tryptophan metabolism, arginine biosynthesis, cysteine and methionine metabolism, and glycerophospholipid metabolism, all of which were found to be activated. Subsequently, non-targeted metabolomic analysis revealed that these differential metabolites were primarily involved in tryptophan metabolism, arginine biosynthesis, cysteine and methionine metabolism, and glycerophospholipid metabolism. Therefore, supplementing PCA in the feed of late-laying hens can improve antioxidant capacity, inflammatory response, and intestinal microbiota composition to enhance intestinal health.

## 1 Introduction

Eggs are an important source of high-quality dietary protein and a major commodity in the global poultry industry. To meet the steadily increasing market demand, the laying hen industry has continuously sought to improve production efficiency, with one key strategy being the extension of the laying cycle to increase total egg output (Arulnathan et al., 2024). However, as hens age, progressive deterioration in intestinal barrier function becomes increasingly evident, as reflected by reduced intestinal villus height, increased intestinal permeability, and downregulated expression of tight junction proteins. Meanwhile, the anti-inflammatory capacity of hens during the late-laying period is significantly reduced, as evidenced by elevated levels of pro-inflammatory cytokines and decreased levels of anti-inflammatory cytokines (Hou et al., 2025; Nii et al., 2025). In addition, laying hens in the late-laying period are in a state of systemic oxidative stress, with decreased antioxidant enzyme activities, related genes, and pathway function, leading to a decline in the egg production rate (Wang et al., 2024). These problems have become major bottlenecks restricting the increase in egg production by extending the egg-laying period.

Intestinal health plays a crucial role in regulating the growth performance, nutrient utilization, and immune function of poultry. The integrity of the intestinal structure enhances the efficiency of poultry in digesting and absorbing nutrients and improves feed conversion efficiency (FCR) (Wickramasuriya et al., 2022). In addition to intestinal morphology, the gut microbiota exerts a profound influence on digestive function by regulating nutrient metabolism, growth, and reproduction through enhancing digestive enzyme activity, producing beneficial metabolites, and inhibiting the proliferation of pathogenic bacteria (Zhang et al., 2025). Meanwhile, as an important component of the immune system, the intestinal microbiota plays a crucial role in regulating host immunity and enhancing disease resistance. Once the intestinal microbiota becomes imbalanced, it may disrupt intestinal barrier function and induce inflammatory responses, thereby reducing production performance (Abd El-Hack et al., 2022). The intestinal health of poultry is also closely related to reproductive performance and growth and development by influencing nutritional metabolism and the establishment of the early intestinal microbiota (Elokil et al., 2022).

PCA is a naturally occurring phenolic acid widely distributed in plant-derived foods and traditional Chinese herbal medicines. It has attracted much attention due to its multiple biological activities, such as antioxidant, anti-inflammatory, and antibacterial activities, as well as its participation in the regulation of lipid and glucose metabolism (Scazzocchio et al., 2015; Yang et al., 2023). Previous studies have shown that PCA can improve intestinal morphology by increasing the height of the intestinal villi and the crypt depth, thereby enhancing the intestinal capacity for nutrient absorption (Wang Y. et al., 2019). In addition, PCA regulates the composition and structure of the intestinal microbiota in broilers. By increasing microbial diversity and maintaining microbial homeostasis, PCA promotes the proliferation of beneficial bacteria while suppressing the growth of potentially pathogenic microorganisms, thereby improving the intestinal microecological environment and supporting gut health. Collectively, these characteristics indicate that PCA may be a promising functional feed additive that can be used to improve the health status and production performance of poultry (Wang Y. et al., 2019).

The existing literature has insufficient exploration of the regulation strategies of PCA components in the feed of late-laying hens, especially in clarifying the relationship between the influence of PCA and the intestinal function of late-laying hens. Therefore, this study aimed to investigate the effects of different PCA concentrations on the intestinal microbiota, transcriptome, antioxidant capacity, and intestinal metabolites of late-laying hens.

## 2 Materials and methods

### 2.1 Animals

A total of 576 Hy-Line Brown laying hens aged 500 days and at the same growth stage with similar physiological conditions were selected and randomly assigned to 4 groups, with 6 replicates per treatment and 24 hens per replicate. The four groups included a basal diet group (CK) and three PCA-supplemented groups: the basal diet supplemented with 0.05% PCA (LPCA), 0.1% PCA (MPCA), and 0.2% PCA (HPCA). The experiment consisted of a 1-week adaptation period followed by a 6-week experimental period, for a total duration of 49 days.

### 2.2 Sample collection

At the end of the experiment, six hens from each replicate in each group were randomly selected, and blood was collected from the wing vein to obtain the serum. The ileal and cecal contents were then collected immediately and stored at −80 °C for subsequent analyses.

### 2.3 Serum biochemical index determination

The concentrations of total cholesterol (TC), triglyceride (TG), high-density lipoprotein cholesterol (HDL), low-density lipoprotein cholesterol (LDL), aspartate aminotransferase (AST), and alanine aminotransferase (ALT) in serum samples were measured following the manufacturer’s instructions (BioSino Bio-Technology & Science Inc., Beijing, China). Commercial assay kits for superoxide dismutase (SOD), catalase (CAT), glutathione peroxidase (GSH-Px), malondialdehyde (MDA), TNF-α, IL-1β, and IL-6 were also used to determine the antioxidant capacity and inflammatory cytokine levels and were supplied by the Beijing Huaying Biotechnology Research Institute.

### 2.4 Transcriptome sequencing and analysis

Total RNA was extracted from ileal tissue samples using QIAzol Lysis Reagent (Qiagen, Germany). RNA concentration and purity were measured using the NanoDrop 2000 spectrophotometer; integrity was evaluated using agarose gel electrophoresis, and the RQN value was determined using the Agilent 5,300 Fragment Analyzer. For library construction, a total RNA amount of ≥30 ng/μL, an RQN of >6.5, and an OD260/280 ratio between 1.8 and 2.2 were required. mRNA was enriched using Oligo(dT) magnetic beads, fragmented, and then reverse transcribed into cDNA. After adapter ligation, fragment screening and library enrichment were carried out. The libraries were sequenced in both directions on the Illumina NovaSeq X Plus platform. The raw data were quality-controlled using Fastp and SeqPrep, and clean reads were obtained. The reads were then aligned to the chicken reference genome (Gallus_gallus) using HISAT2, and gene expression quantification was performed using RSEM. The differential expression analysis was performed using the DESeq2 software, with the criteria of |log2FoldChange| ≥ 1 and Padj<0.05 being adopted for screening. Functional annotation and enrichment analysis were performed based on the Gene Ontology (GO) and Kyoto Encyclopedia of Genes and Genomes (KEGG) databases.

### 2.5 Untargeted Metabolomic analysis

Untargeted metabolomic profiling was performed using 12 chicken cecal content samples, including six biological replicates from the CK group and six biological replicates from the LPCA group. One hen was randomly selected from each replicate for sample collection. The samples were analyzed using liquid chromatography–tandem mass spectrometry (LC–MS/MS) by Majorbio Bio-Pharm Technology Co., Ltd. (Shanghai, China). Quality control (QC) samples were prepared by pooling equal aliquots from all individual samples and were inserted into the analytical sequence to monitor system stability and data quality. Data acquisition was performed in both positive and negative ion modes. The raw mass spectrometry data were processed for peak extraction, alignment, and integration. Subsequently, a series of preprocessing steps were performed, including missing value filtering and imputation, normalization to reduce systematic variation, and evaluation of relative standard deviation (RSD) based on QC samples. Metabolic features with an RSD greater than 30% in QC samples were typically excluded from further analysis. Metabolites were identified by matching MS/MS spectra with those in public databases, including the Human Metabolome Database (HMDB) and METLIN, as well as the company’s in-house library. The identified metabolites were further annotated with KEGG compound IDs for subsequent pathway analysis. Principal component analysis (PCA) was performed as a multivariate statistical method to visualize the overall distribution of samples, while partial least squares discriminant analysis (PLS-DA) was used to maximize the separation between the predefined groups. The robustness of the model was evaluated using cross-validation and permutation tests. The importance of each metabolite in group discrimination was determined based on the variable importance in projection (VIP) scores derived from the PLS-DA model. By integrating the results of univariate and multivariate statistical analyses, significantly different metabolites were identified based on the criteria of |log₂ (fold change) | ≥ 1, *p* < 0.05, and VIP > 1. The differential metabolites were then subjected to hierarchical clustering analysis to visualize their expression patterns, and functional enrichment analysis was performed using the KEGG database to identify significantly enriched metabolic pathways.

### 2.6 Analysis of the intestinal microbiota

Genomic DNA was extracted from the contents of the chicken cecum, and DNA quality was assessed using 1% agarose gel electrophoresis. Specific primers with barcodes were used for polymerase chain reaction (PCR) amplification of the target region to ensure a low and consistent number of cycles. The bacterial 16S rRNA gene was amplified using primers 338F (5′-ACTCCTACGGGAGGCA GCAG-3′) and 806R (5′-GGACTACHVGGGTWTCTAAT-3′). PCR was performed using TransStart FastPfu DNA polymerase and an ABI GeneAmp® 9,700 thermal cycler. The PCR products were verified by 2% agarose gel electrophoresis, purified using the AxyPrep DNA gel recovery kit, and quantified using the QuantiFluor™-ST fluorescence quantitative system. Library construction included adapter ligation, gel purification, denaturation to single-stranded DNA, and cluster amplification on a flow cell (TruSeq™ DNA Sample Prep Kit). Pair-end sequencing was performed on the NextSeq 2000 platform. The raw sequences were quality-filtered, assembled, and noise-reduced using DADA2/Deblur to generate amplicon sequence variants (ASVs). Downstream bioinformatics analysis included taxonomic annotation, alpha and beta diversity analyses, hierarchical clustering, principal component analysis (PCA), principal coordinate analysis (PCoA), non-metric multidimensional scaling analysis (NMDS), and differential abundance analysis (LEfSe) to identify microbial biomarkers and community structure differences.

### 2.7 Data analysis

Statistical analyses of serum biochemical parameters and antioxidant indicators were performed using IBM SPSS Statistics 25.0 (IBM Corp., Armonk, NY, USA). Data are presented as mean ± standard deviation (SD). Differences among the four treatment groups were evaluated using one-way analysis of variance (ANOVA), followed by Duncan’s multiple range test for *post-hoc* comparisons. Statistical significance was defined as a *p*-value of less than 0.05. Microbiota–metabolite and microbiota–gene associations were explored using the Majorbio Cloud Platform^1^ based on Spearman’s correlation analysis, with |r| > 0.6 and *p* < 0.05 considered significant.

## 3 Results

### 3.1 Effects of the PCA on the biochemical indicators and inflammatory cytokines

The results of the serum biochemical indicators showed that there were no significant differences in TC, TG, HDL, LDL, AST, and ALT between the CK group and the PCA groups (Table 1). The inflammatory cytokines, however, showed some significant differences between the CK and PCA groups. TNF-α was significantly decreased in the LPCA and HPCA groups compared with the CK group. The level of IL-1β in the CK group was significantly higher than those in all PCA treatment groups, while IL-6 was significantly higher in the CK group than in the LPCA and HPCA groups (Table 2).

**Table 1:** Effects of different dosages of PCA on biochemical indicators.

Indicators	Groups of different dosages of PCA			
	CK	LPCA	MPCA	HPCA
TC (mmol/L)	2.92 ± 0.9	2.3 ± 0.84	3.06 ± 1.03	2.54 ± 0.67
TG (mmol/L)	17.15 ± 7.18	10.7 ± 5.45	16.81 ± 8.27	14.72 ± 6.56
HDL (mmol/L)	1.94 ± 0.27	1.52 ± 0.59	1.98 ± 0.45	1.68 ± 0.44
LDL (mmol/L)	0.79 ± 0.47	0.5 ± 0.25	0.84 ± 0.54	0.67 ± 0.27
AST (U/L)	253.4 ± 44.35	316.6 ± 78.72	269.94 ± 15.69	272.61 ± 24.57
ALT (U/L)	26.94 ± 45.32	3.32 ± 2.74	18.12 ± 21.97	27.25 ± 34.05

**Table 2:** Effects of different dosages of PCA on inflammatory cytokines.

Indicators	Groups of different dosages of PCA			
	CK	LPCA	MPCA	HPCA
TNF-α (pg/mL)	69.46 ± 15.02^a^	51.71 ± 5.51^b^	55.49 ± 6.18^ab^	51.54 ± 10.33^b^
IL-1β (pg/mL)	33.29 ± 3.8^a^	24.49 ± 1.18^b^	27.32 ± 2.11^b^	23.99 ± 1.46^b^
IL-6 (pg/mL)	168.41 ± 16.55^a^	126.88 ± 9.04^c^	158.82 ± 5.75^ab^	143.17 ± 12.84^bc^

Different superscript letters indicate that the indicator has significant differences among different treatment groups (*p* < 0.05).

### 3.2 Effects of PCA on the antioxidant capacity

In terms of the antioxidant capacity, the LPCA group showed significant differences compared with the other groups. The three indicators of SOD, CAT, and GSH-Px were all highest in the LPCA group, showing significant differences compared with all other groups. In contrast, MDA was lowest in the LPCA group and also differed significantly from the other groups (Table 3).

**Table 3:** Effects of different dosages of PCA on antioxidant capacity.

Indicators	Groups of different dosages of PCA			
	CK	LPCA	MPCA	HPCA
SOD (U/L)	61.43 ± 3.41^c^	82.06 ± 4.66^a^	66.51 ± 4.64^c^	74.76 ± 2.99^b^
CAT (U/L)	34.89 ± 4.29	49.98 ± 5.68^a^	40.93 ± 4.19^bc^	43.28 ± 3.37^ab^
GSH-PX (U/L)	134.42 ± 18.1^c^	178.63 ± 17.97^a^	152.89 ± 5.81^bc^	161.61 ± 8.1^ab^
MDA (mmol/L)	3.86 ± 0.57^a^	2.24 ± 0.42^c^	3.13 ± 0.23^b^	2.9 ± 0.38^bc^

Different superscript letters indicate that the indicator has significant differences among different treatment groups (*p* < 0.05).

### 3.3 Transcriptome sequencing analysis of ileum tissue between the CK group and the LPCA group

To understand the differences in the transcriptome between the control group and the treatment group, we performed RNA sequencing on the ileal tissues of the CK group and the LPCA group. Principal component analysis showed that the two groups were divided into two different clusters, indicating significant differences between the CK group and the LPCA group (Figure 1A).

**Figure 1:**
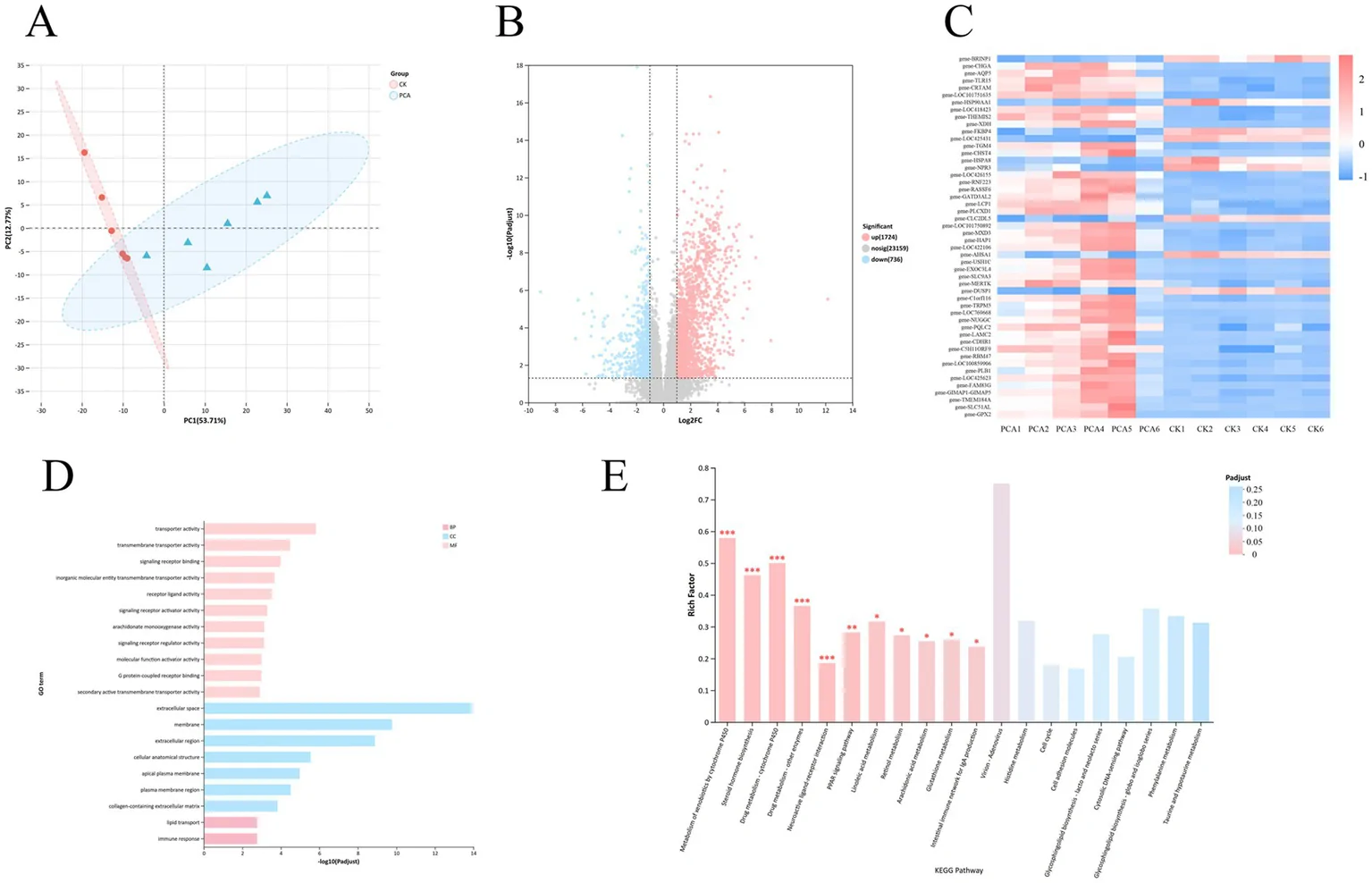
Effects of PCA on the intestinal transcriptome of late-laying hens of the CK and LPCA groups. **(A)** Principal component analysis score plot. **(B)** Volcano plot of DEGs. **(C)** Heatmap made by standardizing the expression levels of the 50 differentially expressed genes with the smallest *p*-values. **(D)** Bar graph of the GO enrichment analysis. **(E)** Bar graph of the KEGG enrichment analysis.

A total of 2,460 differentially expressed genes (DEGs) were identified, including 1,724 upregulated genes and 736 downregulated genes (Figure 1B). Hierarchical cluster analysis was then performed on the DEGs, and the expression levels of the 50 genes with the smallest *p*-values were standardized and visualized as a heatmap (Figure 1C). To further elucidate the biochemical functions of differentially expressed genes (DEGs), GO and KEGG enrichment analyses were performed. A total of 1,679 genes were successfully annotated with GO terms. Among these, 532 DEGs were significantly enriched in biological processes (BP), 87 DEGs were associated with cellular components (CC), and 241 DEGs were involved in molecular functions (MFs).

The enriched biological processes primarily included transporter activity, transmembrane transporter activity, signaling receptor binding, inorganic molecular entity transmembrane transporter activity, receptor ligand activity, signaling receptor activator activity, arachidonate monooxygenase activity, signaling receptor regulator activity, molecular function activator activity, G protein-coupled receptor binding, and secondary active transmembrane transporter activity. Additionally, cellular components include extracellular space, membrane, extracellular region, cellular anatomical structure, apical plasma membrane, plasma membrane region, and collagen-containing extracellular matrix, while molecular functions include lipid transport and immune response (Figure 1D).

KEGG analysis indicated that DEGs were mainly enriched in the metabolism of xenobiotics by cytochrome P450, steroid hormone biosynthesis, drug metabolism-cytochrome P450, neuroactive ligand–receptor interaction, the PPAR signaling pathway, linoleic acid metabolism, retinol metabolism, arachidonic acid metabolism, glutathione metabolism, and intestinal immune network for IgA production (Figure 1E).

### 3.4 Effects of PCA on intestinal microbiota

After analyzing the serum biochemical indicators, we found that the LPCA group showed the most favorable results; therefore, we analyzed the cecal microbial communities in the CK and LPCA groups to understand the impact of PCA on the microbial structure in the cecum. The results of the alpha diversity analysis showed that, compared with the CK group, at the species level, the four indices—Shannon, Sobs, Chao1, and Ace—were significantly lower in the LPCA group at the species level (*p* < 0.05) (Figure 2A). PCoA analysis revealed significant differences in the composition of the cecum microbiota between the CK group and the LPCA group (Figure 2B). The LEfSe method was used to identify taxa with significant differences between the CK and LPCA groups (*p* < 0.05, LDA score> 2). The main taxa enriched in the CK group were c_Actinobacteria, c_Bacilli, o_Bifidobacteriale, o_Eubacteriales, o_Lactobacillales, f_Bifidobacteriaceae, f_Lactobacillaceae, f_Tannerellacea, g_Aeriscardovia, g_Alistipes, g_Lactobacillus, g_Ligilactobacillus, and g_Limosilactobacillus, whereas the main taxa enriched in the LPCA group were f_Bacteroidaceae, f_Marinifilaceae, g_Bacteroides, g_Faecalibacterium, and g_Odoribacter (Figures 2C,D).

**Figure 2:**
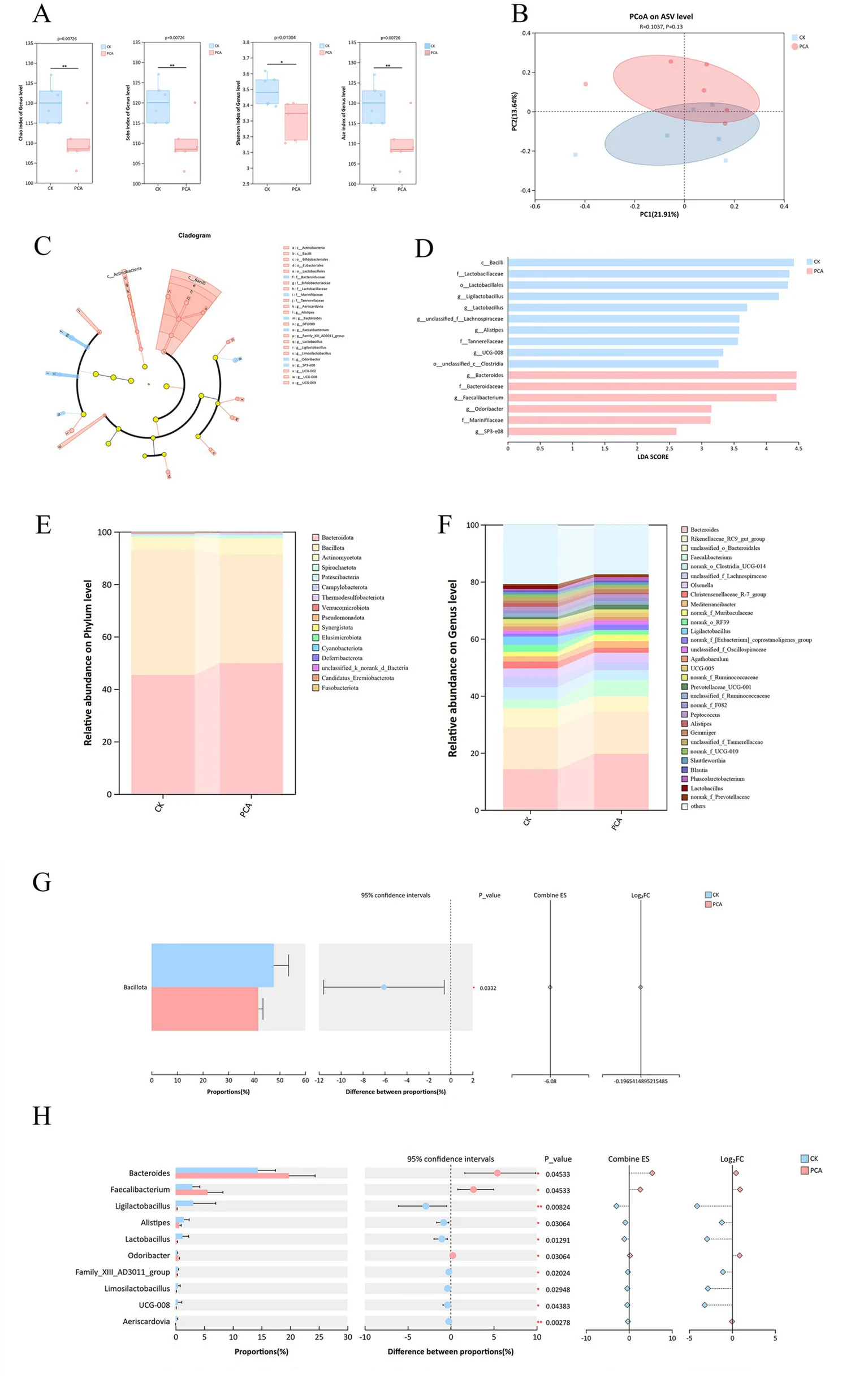
Effects of PCA on the intestinal microbiota of late-laying hens. **(A)** Boxplots of alpha diversity indices of the CK and LPCA groups. **(B)** PCoA plot of ASV levels of the CK and LPCA groups. **(C)** LEfSe cladogram of the CK and LPCA groups. **(D)** LDA bar chart of the CK and LPCA groups. **(E)** Taxa composition plot of the CK and LPCA groups at the phylum level. **(F)** Taxa composition plot of the CK and LPCA groups at the genus level. **(G)** Species difference analysis plot of the CK and LPCA groups at the phylum level. **(H)** Species difference analysis plot of the CK and LPCA groups at the genus level.

Compared with the CK group, at the phylum level, Bacillota was significantly lower in the LPCA group; at the genus level, Bacteroides, Mediterraneibacter, Odoribacter, Acutalibacter, and SP3-e08 were significantly higher, while Lactobacillus and Aeriscardovia were significantly lower (Figures 2E–H).

### 3.5 Effects of PCA on the intestinal metabolites

We also analyzed the metabolites in the cecal contents of the CK and LPCA groups. The results are shown in Figure 3. Partial least squares discriminant analysis (PLS-DA) in both positive and negative ion modes showed differences in the composition of cecal metabolites between the CK group and the LPCA group (Figures 3A,B). Subsequent permutation tests confirmed that the model exhibited good fit and predictive ability, and no outliers were observed (Figures 3C,D). A total of 264 differential metabolites were identified in this analysis. Among them, 59 metabolites were upregulated, and 39 were downregulated in the positive ion mode, while 102 metabolites were upregulated, and 64 were downregulated in the negative ion mode (Figures 3E–G). KEGG analysis indicated that these metabolites were significantly enriched in the following pathways: biosynthesis of cofactors; autophagy; nucleotide metabolism; arginine biosynthesis; efferocytosis; tryptophan metabolism; D-amino acid metabolism; cysteine and methionine metabolism; ABC transporters; pantothenate and CoA biosynthesis; phenylalanine, tyrosine, and tryptophan biosynthesis; sulfur metabolism; and glycerophospholipid metabolism pathways (Figure 3H).

**Figure 3:**
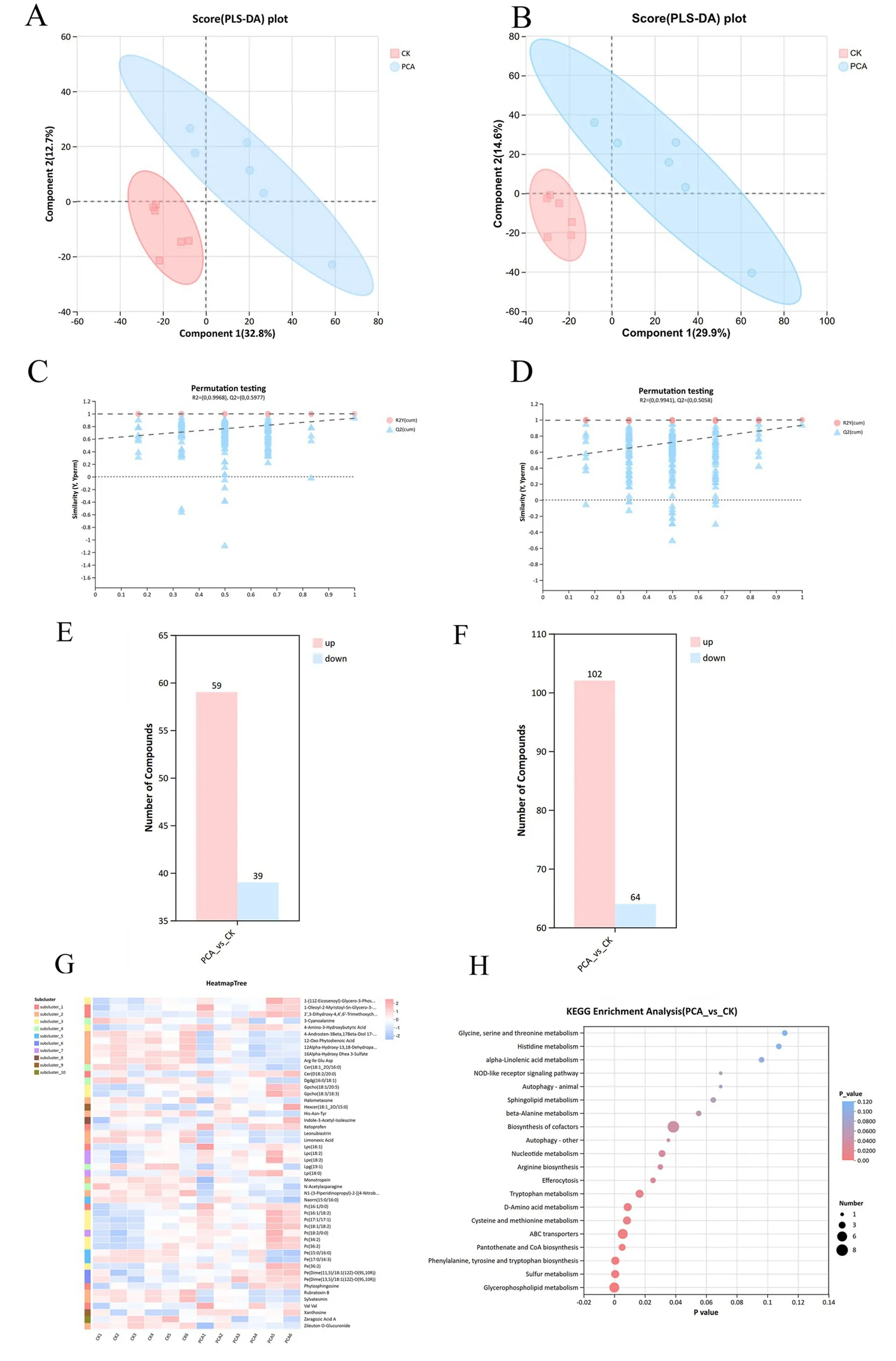
Effects of PCA on the intestinal metabolites of late-laying hens. **(A)** PLS-DA score plot of the CK and LPCA groups in the positive mode. **(B)** PLS-DA score plot of the CK and LPCA groups in the negative mode. **(C)** Model interpretability (R2Y) and predictability (Q2) in the positive mode. **(D)** Model interpretability (R2Y) and predictability (Q2) in the negative mode. **(E)** Differential substance screening chart of the CK and LPCA groups in the positive mode. **(F)** Differential substance screening chart of the CK and LPCA groups in the negative mode. **(G)** Clustering heatmap of differential substance enrichment analysis of the CK and LPCA groups. **(H)** Bubble plot of KEGG enrichment analysis of the CK and LPCA groups. The horizontal axis represents the *p*-value for enrichment significance. The smaller the *p*-value, the greater the statistical significance. The vertical axis represents the KEGG pathway. The size of the bubbles represents the number of metabolites enriched in each pathway.

### 3.6 Integrated analysis of microbiome and transcriptomics

Based on the correlation analysis, Ligilactobacillus showed significant positive correlations with the genes COL3A1, COL1A1, MYL6, CSRP1, PCP4, and HSPA2 (Figures 4A,B). Mantel test analysis revealed a significant positive correlation between the gut microbial community structure and GPX2 gene expression in the PCA-treated group (Figure 4C).

**Figure 4:**
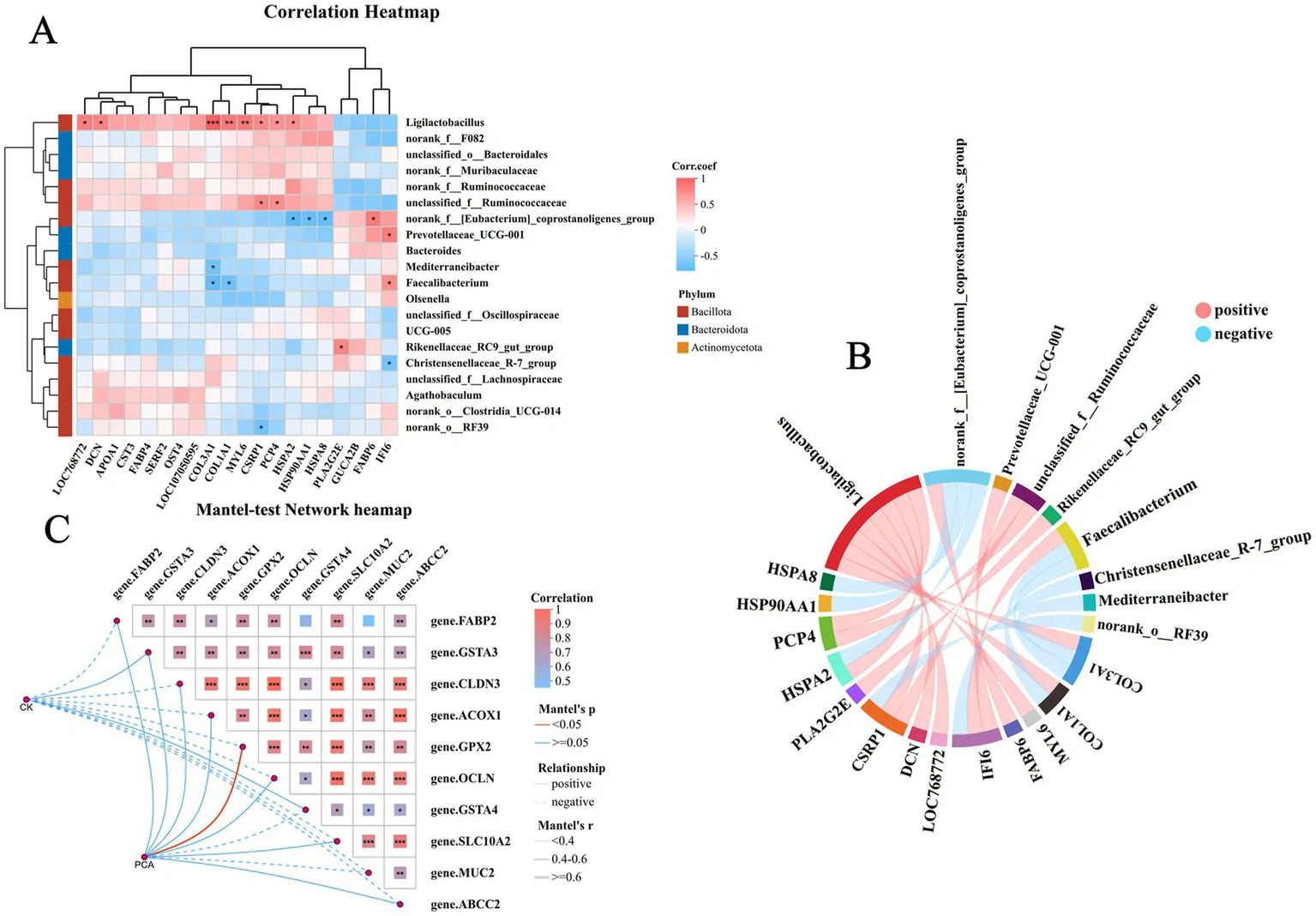
Integrated analysis of microbiome and transcriptomics. **(A)** Heatmap of clustered correlations between differential microorganisms and differential genes in the top 20 (*p* < 0.05). **(B)** Chord diagrams between differential microbes and differential genes in the top 20 (*r*^2^ > 0.6 and *p* < 0.05).**(C)** Mantel test analysis plot of microorganisms and genes. The lines represent the Mantel correlation relationships between the microbial community matrix and metabolite matrix. Solid lines indicate positive correlations, whereas dashed lines indicate negative correlations. The color intensity represents the strength of the correlation. Asterisks in the colored blocks indicate statistical significance, with * representing 0.01 < *p* ≤ 0.05, ** representing 0.001 < *p* ≤ 0.01, and *** representing *p* ≤ 0.001. The line width represents the absolute value of Mantel’s r, with thicker lines indicating stronger correlations between the two matrices.

### 3.7 Integrated analysis of microbiome and metabolomics

Correlation analysis was further performed to evaluate the associations between differential bacterial genera and differential metabolites (Figures 5A,B). The results showed that several bacterial genera exhibited significant correlations with metabolites at the genus level. Specifically, Bacteroides was significantly positively correlated with Lpc (18:2), and Faecalibacterium showed a significant positive correlation with Pc (16:1/0:0). In addition, Christensenellaceae_R-7_group exhibited a significant negative correlation with 1-oleoyl-2-myristoyl-Sn-glycero-3-phosphocholine. Mantel test analysis revealed a significant positive correlation between the gut microbial community structure and the Dgdg (16:0/18:2) metabolism in the CK group (Figure 5C).

**Figure 5:**
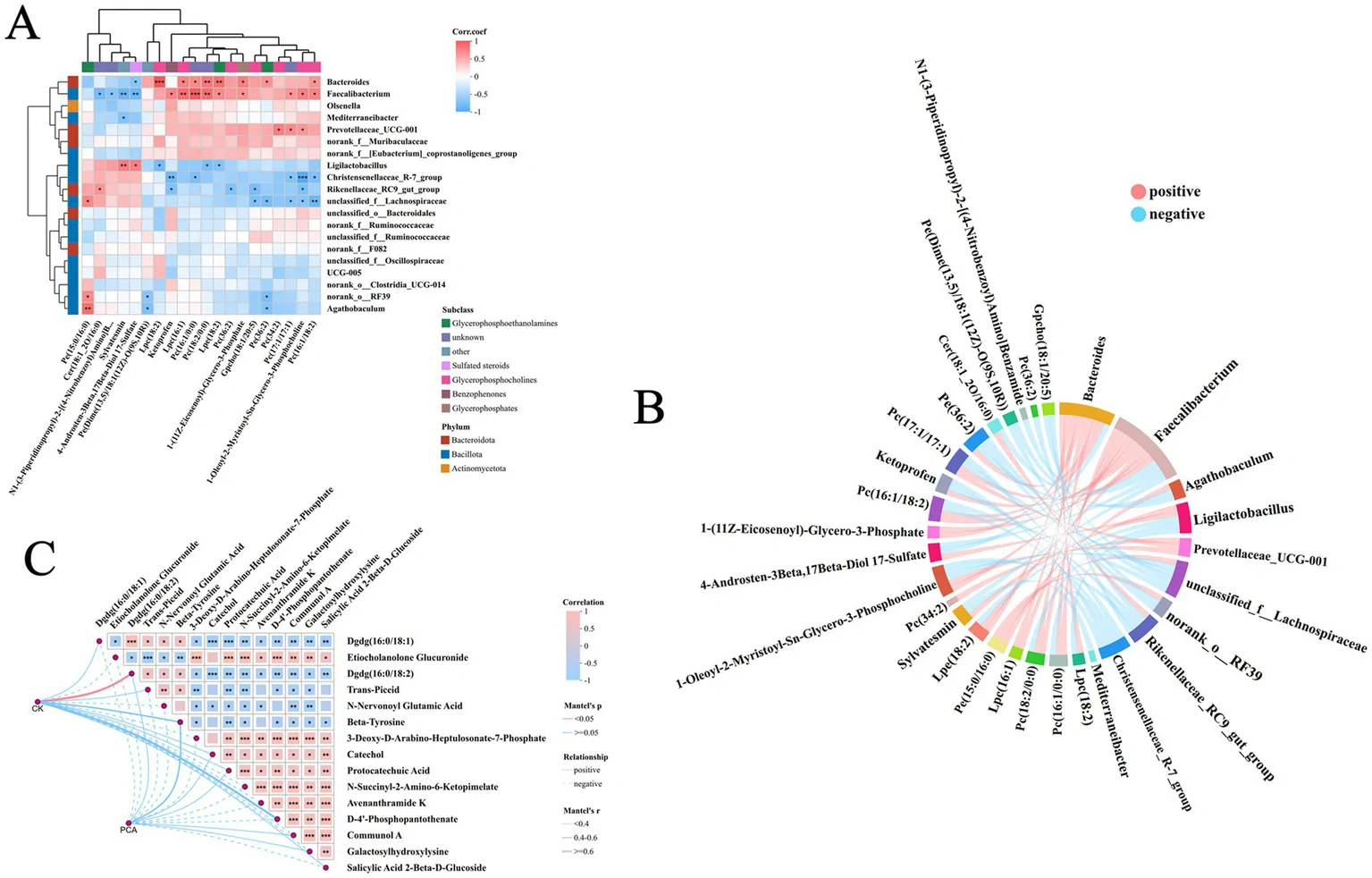
Integrated analysis of microbiome and metabolomics. **(A)** Heatmap of clustered correlations between differential microorganisms and differential metabolites in the top 20 (*p* < 0.05). **(B)** Chord diagrams between differential microbes and differential metabolites in the top 20 (*r*^2^ > 0.6 and *p* < 0.05). **(C)** Mantel test analysis plot of microorganisms and metabolites. The lines represent the Mantel correlation relationships between the microbial community matrix and the metabolite matrix. Solid lines indicate positive correlations, whereas dashed lines indicate negative correlations. The color intensity represents the strength of the correlation. Asterisks in the colored blocks indicate statistical significance, with * representing 0.01 < p ≤ 0.05, ** representing 0.001 < *p* ≤ 0.01, and *** representing *p* ≤ 0.001. The line width represents the absolute value of Mantel’s *r*, with thicker lines indicating stronger correlations between the two matrices.

## 4 Discussion

PCA is a phenolic compound naturally found in fruits and vegetables. In recent years, PCA has received increasing attention in the fields of nutritional science and biochemistry. Emerging evidence suggests that PCA exhibits potent antioxidant properties by scavenging free radicals and enhancing endogenous antioxidant defense systems (Tsao, 2010). In addition, PCA possesses anti-inflammatory properties by regulating key signaling pathways, including the inhibition of pro-inflammatory cytokines (Li H. et al., 2025). Studies have also demonstrated that PCA exerts beneficial effects on gastrointestinal health by modulating the composition of the intestinal microbiota, maintaining intestinal barrier integrity, and promoting nutrient digestion and absorption (Wang Y. et al., 2019). These characteristics indicate that PCA may be a promising feed additive that could improve intestinal health, immune response, and growth performance in animals.

Oxidative stress and inflammatory responses are key factors affecting the intestinal health of poultry. Oxidative stress arises from an imbalance between the production of reactive oxygen species (ROS) and the antioxidant defense system, leading to cellular damage and impaired physiological functions. Superoxide dismutase (SOD) is one of the core members of the enzymatic antioxidant system, with the capacity to convert superoxide anion free radicals into hydrogen peroxide, thereby reducing free-radical -induced cellular damage (Wu et al., 2021). Glutathione peroxidase (GSH-Px) has the ability to eliminate harmful hydrogen peroxide and lipid peroxides by converting them into less harmful substances using reduced glutathione, thereby protecting cells from oxidative damage (Cheng et al., 1999). Catalase (CAT) is a key antioxidant enzyme that catalyzes the decomposition of hydrogen peroxide and plays an important role in maintaining intracellular redox homeostasis. Together with SOD and GSH-Px, CAT constitutes a major enzymatic defense system against oxidative stress (Birben et al., 2012). Malondialdehyde (MDA) is the end product of lipid peroxidation and a marker of oxidative stress. The increase in its level is usually proportional to the degree of oxidative damage to cells (Ruottinen et al., 2020). This study found that PCA supplementation significantly increased the activities of antioxidant enzymes, including SOD, CAT, and GSH-Px, while simultaneously reducing MDA levels. These effects were particularly pronounced in the LPCA group, suggesting that PCA effectively alleviated oxidative damage and enhances antioxidant capacity. Previous studies have also shown that PCA can significantly reduce the levels of IL-6, IL-2, and TNF-α, thereby demonstrating anti-inflammatory effects (Hu et al., 2020). Similarly, our results showed that PCA supplementation significantly decreased the levels of the pro-inflammatory cytokines TNF-α, IL-1β, and IL-6. Together with previous evidence, these findings provide further support for the potential anti-inflammatory properties of PCA in late-laying hens.

The intestinal microbiota plays a pivotal role in maintaining intestinal homeostasis, nutrient metabolism, and immune regulation. Our results showed that PCA significantly altered the composition and structure of the cecal microbiota; although the α-diversity indices decreased, PCoA analysis indicated that the structure of the gut microbiota had undergone significant changes. We found that the LPCA group was enriched with beneficial bacterial communities, such as g_Bacteroides and g_Odoribacter. Considering the biological characteristics of PCA, the reduction in α-diversity indices may be partly attributed to its selective regulatory effects on specific bacterial populations. Integrated analysis of microbial diversity and metabolomics further revealed that Lpc (18:2), a metabolite enriched in the glycerophospholipid metabolism pathway, was significantly positively correlated with the genus Bacteroides. Metabolomic analysis further showed that the level of Lpc (18:2) was significantly increased in the PCA group compared with the CK group. Therefore, we speculate that PCA may reshape the gut microbial community by regulating the intestinal metabolic environment, which may facilitate the enrichment of specific beneficial taxa and contribute to gut microbial homeostasis. However, it should be noted that the above interpretation is based on the experimental results obtained in the present study, including the inferred associations from the correlation analysis between microbiota and metabolites. The causal relationship between PCA-induced metabolic changes and the selective enrichment of specific bacterial taxa requires further functional validation.

g_Bacteroides is a core symbiotic microbiota in the intestinal tract, oral cavity, and other parts of humans and animals (Shin et al., 2024). It can maintain microecological balance and degrade complex polysaccharides to produce short-chain fatty acids (SCFAs), such as acetic acid and propionic acid, which are crucial for the development of the host’s immune system and metabolic health (Shin et al., 2024). g_Odoribacter can synthesize a variety of SCFAs, playing a crucial role in promoting intestinal health, enhancing intestinal barrier function, supporting energy metabolism, and regulating host immune responses (Li J. et al., 2025). The increased abundance of these two types of microbiota in the PCA treatment group suggests that PCA can contribute to intestinal health by regulating the production of short-chain fatty acids.

In addition, the Mantel test results from the integrated analysis of microbial diversity and transcriptomics showed that GPX2 expression was not significantly correlated with gut microbial community structure in the CK group, whereas a significant positive correlation was observed after PCA treatment. Combined with the transcriptomic analysis showing a significant upregulation of GPX2 expression, these results indicate that PCA treatment not only increased GPX2 transcriptional expression but also strengthened the association between the gut microbial community structure and GPX2 expression. As an important intestinal antioxidant enzyme, GPX2 catalyzes the reduction of hydrogen peroxide and lipid peroxides through glutathione-dependent reactions, thereby maintaining intestinal redox homeostasis. Therefore, these results suggest that PCA-induced gut microbial remodeling may occur in coordination with GPX2-mediated antioxidant defense, jointly contributing to the maintenance of intestinal redox homeostasis. However, the Mantel test only reflects the correlation between microbial community structure and host gene expression and cannot demonstrate a direct causal relationship between them. The specific regulatory mechanisms require further investigation.

Metabolomic analysis further supported the regulatory effect of PCA on intestinal function. The differential metabolites were predominantly enriched in pathways closely associated with intestinal digestion and absorption, including tryptophan metabolism, arginine biosynthesis, cysteine and methionine metabolism, and glycerophospholipid metabolism, all of which showed significant alterations following PCA supplementation. Among these pathways, tryptophan metabolism plays a crucial role in regulating gastrointestinal motility, secretion, blood vessel widening, and nutrient absorption through the serotonin pathway, microbial indole pathway, and kynurenine pathway. It also supports the integrity of the intestinal barrier and mucosal balance and helps control local immune responses and mucosal protection (Niu et al., 2025). Arginine biosynthesis promotes intestinal epithelial repair through the SLC7A2 transporter, maintains intestinal barrier function, and regulates immune responses through polyamine metabolic pathways. Additionally, arginine metabolism interacts closely with the gut microbiota and has been implicated in the regulation of inflammatory responses and immune tolerance (Niu et al., 2025). Cysteine, a key metabolite in methionine metabolism, plays an important role in maintaining intestinal health. It contributes to redox homeostasis and intracellular signaling in intestinal epithelial cells and is involved in the regulation of cell proliferation and intestinal barrier integrity (Burrin and Stoll, 2007). In addition, this pathway is also highly active in the intestinal microbiota; some bacteria in the intestinal tract can utilize cysteine and methionine as substrates for sulfur cycling and growth. The microbial metabolism of these amino acids may contribute to the host’s digestion and nutrient utilization (Kim et al., 2024). Glycerophospholipid metabolism serves as a key carrier of essential nutrients such as choline and polyunsaturated fatty acids, and participates in the assembly and transport of chylomicrons (Wang T. et al., 2019). Meanwhile, this metabolic process is involved in the regulation of intestinal function by affecting cholesterol absorption and modulating alkaline sphingomyelinase activity (Liu et al., 2000; Nilsson et al., 2021). These findings suggest that PCA may contribute to improved intestinal health by modulating intestinal epithelial barrier integrity, nutrient absorption, and microbial metabolic processes. Subsequent transcriptomic analyses further elucidated the mechanisms by which PCA may modulate intestinal health. Gene Ontology (GO) enrichment analysis revealed significant enrichment of biological functions related to transporter activity, transmembrane transporter activity, and secondary active transmembrane transporter activity, suggesting that PCA significantly affected the transmembrane transport of ions, nutrients, and exogenous substances in the ileum. The ABC and SLC family transporters highly expressed in the intestinal tract not only participate in nutrient absorption but also mediate the excretion of bacterial metabolic products. Their activity is closely related to intestinal homeostasis (Kumar et al., 2015). GO-related cellular components, including extracellular regions, membrane structures, and the collagen-containing extracellular matrix (ECM), were also significantly enriched. These structures constitute key components of the intestinal barrier because they physically separate the intestinal internal environment from the intestinal immune system, preventing inflammation caused by the activation of the immune system from affecting digestion and absorption (Neurath et al., 2025). The enrichment of membrane and extracellular matrix (ECM)-related components suggests that the structural integrity of the intestinal barrier is enhanced, which is crucial for preventing excessive permeability and inflammation. In addition, molecular functions related to receptor binding, receptor ligand activity, and G protein-coupled receptor (GPCR) binding were also significantly enriched. These signaling pathways play a core role in regulating the interaction between the host and the microbiota. Microbial metabolites such as SCFAs, bile acids, and tryptophan-derived compounds act as ligands that bind to receptors on the host cell surface, particularly the GPCR family members, such as GPR41/43 and TGR5, thereby transmitting signals from the gut microbiota to the host and modulating inflammatory responses and intestinal barrier function (Fu et al., 2023).

KEGG analysis revealed that differentially expressed genes (DEGs) were largely enriched in pathways closely related to the digestive system, such as the PPAR signaling pathway and glutathione metabolism, with significant upregulation of genes including FABP2, ACOX1, GSTA3, and GSTA4, indicating the activation of these pathways in the PCA-treated group. Studies have shown that the PPAR signaling pathway is not only involved in regulating the processing and transport of long-chain unsaturated fatty acids in the intestinal epithelium but also in regulating lipid metabolism (Duszka et al., 2017). In addition, PPARα can regulate the expression of genes related to lipid and energy metabolism; PPARβ is involved in cell proliferation and lipid uptake; and PPARγ is highly expressed in the proximal part of the small intestine, responding to the activation of long-chain unsaturated fatty acids (Vasques-Monteiro et al., 2024). These functions position the PPAR signaling pathway as a crucial hub linking lipid digestion and intestinal homeostasis. Regarding glutathione metabolism, research has shown that oxidized glutathione ingested by the body can be reduced to reduced glutathione in the upper part of the small intestine and then absorbed and transported to the liver. This mechanism—making glutathione—not only participates in maintaining the REDOX balance in the intestinal tract but also contributes to the transport of dietary antioxidant components (Yamada et al., 2018).

Taken together, PCA-induced alterations in the gut microbiota may be associated with changes in intestinal metabolite profiles, which may subsequently influence host gene expression and physiological functions. These findings provide a potential multi-layered framework for understanding the mechanisms underlying the beneficial effects of PCA on intestinal health.

## 5 Conclusion

Taken together, we demonstrated that dietary PCA supplementation at a concentration of 0.05% enhanced antioxidant capacity and modulated the immune response in late-laying hens. The underlying mechanism may include PCA’s antioxidant effects, modulation of the intestinal microbiota, and maintenance of the intestinal epithelial barrier integrity. This study may provide a potential nutritional strategy for improving intestinal health in late-laying hens.

## Data Availability

The datasets generated for this study can be found in the NCBI https://www.ncbi.nlm.nih.gov/Traces/study/?acc=PRJNA1455433.

## Footnotes

1
www.majorbio.com
